# Mapping evidence on HIV status awareness among key and vulnerable populations in sub-Sahara Africa: a scoping review protocol

**DOI:** 10.1186/s13643-020-01537-w

**Published:** 2020-11-24

**Authors:** Clement Avoka, Patience Adzordor, Vitalis Bawontuo, Diana A. Akila, Desmond Kuupiel

**Affiliations:** 1 Faculty of Health and Allied Sciences, Catholic University College of Ghana, Fiapre, Sunyani, Ghana; 2 Research for Sustainable Development Consult, Sunyani, Ghana; 3 University of Health and Allied Sciences, Ho, Ghana; 4 Department of Public Health Medicine, School of Nursing and Public Health, University of KwaZulu-Natal, Durban, 4001 South Africa

**Keywords:** UNAIDS 90:90:90, HIV/AIDS, Vulnerable populations, HIV key populations, HIV status awareness, Sub-Sahara Africa

## Abstract

**Background:**

Human immunodeficiency virus (HIV) infection and acquired immunodeficiency syndrome (AIDS) continue to be a major public health issue, especially in sub-Sahara Africa (SSA). Literature shows significant HIV status awareness, testing, and treatment have generally improved among the population since the inception of the UNAIDS 90:90:90 programme. Despite this, it is possible literature gaps exist that require future research to inform in-country programmes to improve the gains post-UNAIDS 90:90:90 programme. This study, therefore, aims to synthesize literature and describe the evidence on HIV status awareness among key and vulnerable populations in SSA focusing on the first UNAIDS 90 since it is essential for treatment initiation.

**Method:**

This systematic scoping review will be guided by the framework proposed by Arksey and O’Malley and improved by Levac and colleagues. Literature searches will be conducted in PubMed, SCOPUS, CINAHL, Google Scholar, and Science Direct from 2016 to 2020. A snowball approach will also be used to search for relevant articles from the reference of all included studies. This study will include both published and grey literature, articles that include HIV key and vulnerable populations, HIV status awareness, and evidence from SSA countries. Two reviewers will independently conduct the abstract and full-text article screening as well as pilot the data extraction form. Thematic content analysis and a summary of the themes and sub-themes will be reported narratively.

**Discussions:**

The evidence that will be provided by this study may be useful to inform in-country programmes to improve the gains made post-UNAIDS 90:90:90 programme from 2021 onwards. This study also anticipates identifying literature gaps to guide researchers interested in this field of study in the future. Peer review journals, policy briefs, and conference platforms will be used to disseminate this study’s findings.

## Background

The human immunodeficiency virus (HIV) and acquired immunodeficiency syndrome (AIDS) continue to be a major public health issue, particularly in sub-Sahara Africa (SSA) where more than two thirds (25.7 million) of the world’s estimated 39.7 million people living with HIV/AIDS live [1–3]. The risk behaviours and vulnerabilities of specific populations and their networks determine the dynamics of HIV epidemics. HIV key populations such as men who have sex with men, people who inject drugs, people in prisons and other closed settings, sex workers and their clients, and transgender people in almost all settings are disproportionately affected by HIV [2]. Without addressing the needs of these key populations, a sustainable response to HIV will not be achieved [4].

Populations such as adolescent girls and young women may also be vulnerable to HIV infection since research has shown that more than 80% of the HIV-infected adolescents live in sub-Saharan Africa [5]. Global estimates showed that in 2018, nearly 770,000 people died from HIV-related causes and 1.7 million people were newly infected mainly among HIV key and vulnerable populations due to gaps in HIV services [6, 3, 7]. The Joint United Nations Programme on HIV/AIDS (UNAIDS) in its quest to end HIV/AIDS pandemic set the ambitious 90-90-90 target in 2015 [8, 1, 9].

The UNAIDS 90:90:90 programme aims to achieve the following goals by 2020: 90% of all people living with HIV will know their HIV status, 90% of people with diagnosed HIV will receive antiretroviral therapy (ART), and 90% of all people on HIV treatment will achieve viral suppression by catching up in settings with low service coverage [9]. To achieve this, various countries have put in place several strategies that are increasing access to effective HIV prevention, diagnosis, treatment, and care, including opportunistic infections. The efforts by national HIV programmes supported by civil society groups and international development partners are contributing to achieving the UNAIDS 90:90:90 targets [8]. Global estimates showed that about 79% of people living with HIV knew their status, 62% were receiving ART, and 53% had achieved viral suppression at the end of 2018 [8, 1]. Nonetheless, there is a knowledge gap of these UNAIDS 90:90:90 target achievements among each HIV vulnerable and key populations. This knowledge would be essential to help provide targeted HIV services. Therefore, this study will aim to synthesize literature and describe the evidence on HIV status awareness among HIV key populations and vulnerable populations in SSA focusing on the first 90 of the UNAIDS 90:90:90 goals due to its relevance for treatment initiation. The evidence that will be provided by this study may be useful to inform in-country programmes to improve the gains made post-UNAIDS 90:90:90 programme from 2021 onwards. This study also anticipates identifying literature gaps to guide researchers interested in this field of study in the future. The evidence that will be provided by future researches may also guide decisions towards ending the AIDS epidemic by 2030 as stipulated by the United Nations Sustainable Development Goals 3.3 [10].

## Methods

The development of this protocol reasonably followed the preferred reporting item for systematic reviews and meta-analyses extension for a protocol (PRISMA-P) guidelines (Supplementary file 1). However, we will use the PRISMA extension for scoping review checklist to guide the reporting of the results paper. A systematic scoping review study will be conducted to answer the review question. This is because it ensures a systematic search for literature, an examination of the literature, and a description of the range of evidence on a research area of interest [11, 12]. This proposed scoping review study will be conducted in line with the steps proposed by Arksey and O’Malley in their 2005 framework [11]. These steps include identifying the research question, identifying relevant studies, study selection, charting the data, and collating, summarizing, and reporting results [11].

### Identifying the research question

This study will be undertaken to answer the following research question. To date, what evidence exists on HIV status awareness among key and vulnerable populations in SSA? The population, concept, and context (PCC) framework defining the eligibility of this review question is illustrated in Table 1. The sub-research questions will be as follows:

1. To date, what evidence exists on HIV status awareness among men who have sex with men in SSA?
2. To date, what evidence exists on HIV status awareness among prisoners in SSA?
3. To date, what evidence exists on HIV status awareness among sex workers in SSA?
4. To date, what evidence exists on HIV status awareness among people who inject drugs in SSA?
5. To date, what evidence exists on HIV status awareness among transgender people in SSA?
6. To date, what evidence exists on HIV status awareness among adolescents’ girls and young women in SSA?

**Table 1:** PCC framework defining the eligibility of this review question

Population	HIV key populations: These will include men who have sex with men, people in prisons, sex workers, transgender people, and people injecting drugs. Vulnerable populations: These will include adolescents’ girls (10–19 years) and young women (age 15–24 years)
Concept	HIV status awareness: This includes key and populations knowing their HIV status.
Context	Africa: This will include countries in the WHO Africa region (Algeria, Angola, Benin, Botswana, Burkina Faso, Burundi, Cameroon, Cabo Verde, Central African Republic, Chad, Comoros, Congo, Côte d'Ivoire, the Democratic Republic of the Congo, Equatorial Guinea, Eritrea, Ethiopia, Gabon, The Gambia, Ghana, Guinea, Guinea-Bissau, Kenya, Lesotho, Liberia, Madagascar, Malawi, Mali, Mauritania, Mauritius, Mozambique, Namibia, Niger, Nigeria, Rwanda, Sao Tome and Principe, Senegal, Seychelles, Sierra Leone, South Africa, South Sudan, Swaziland, Togo, Uganda, United Republic of Tanzania, Zambia, and Zimbabwe)

### Identifying relevant studies

An in-depth search for relevant peer-reviewed articles published in the following bibliographic databases: PubMed, CINAHL, Google Scholar, Science Direct, and SCOPUS, will be conducted from 1 January 2016 to the last search date in December 2020. The authors will utilize the following keywords: ‘men having sex with men’, ‘gay’ ‘homosexual’, ‘prison’, ‘prisoner’, ‘inmates’, ‘sex workers’, ‘prostitute’, ‘sex workers and clients’, ‘transgender people’, ‘injection drug user’, ‘Adolescent’, ‘Adolescence’, ‘adolescent’, ‘girls’, ‘teen’, ‘child’, ‘children’, ‘young women’, ‘women’, ‘female’, ‘human immunodeficiency virus’, ‘HIV’, ‘acquired immunodeficiency syndrome’, ‘AIDS’, ‘HIV testing’, ‘HIV diagnosis’, ‘status awareness’, ‘positive status awareness’, ‘UNAIDS 90:90:90’, ‘sub-Saharan Africa’, ‘SSA’. We will use a search strategy that employs search terms such as keywords AND/OR and medical subject headings (MeSH) or subject headings that relate to key concepts, combines search terms within a concept with the Boolean term ‘OR’, combines search terms between concepts with the Boolean term ‘AND’, and is adapted to the syntax used by each database. This study will additionally explore the reference lists of all included studies for relevant studies. Limitations on study designs and language of publication will be removed during the search in the databases. However, the date will be customized from 2015 to 2020 since the UNAIDS 90:90:90 programme started in 2016. Where possible, the search will also be limited to humans. A pilot search for this study conducted in PubMed is illustrated in Table 2.

**Table 2:** A pilot search conducted in the PubMed database

Date	Database	Keywords	Search results
11 November 2020	PubMed (2016–2020)	(((((((((((((((((((((((((men having sex with men[MeSH Terms]) OR (sexual and gender minorities[MeSH Terms])) OR (prisoner[MeSH Terms])) OR (inmates[MeSH Terms])) OR (sex worker[MeSH Terms])) OR (prostitute[MeSH Terms])) OR (transgender people[MeSH Terms])) OR (injection drug user[MeSH Terms])) OR (adolescents[MeSH Terms])) OR (teenager[MeSH Terms])) OR (Child[MeSH Terms])) OR (children[MeSH Terms])) OR (girls[MeSH Terms])) OR (women[MeSH Terms])) OR (female[MeSH Terms])) AND (HIV status awareness[MeSH Terms])) OR (human immunodeficiency virus status[MeSH Terms])) OR (positive status awareness[MeSH Terms])) OR (HIV sero-status[MeSH Terms])) OR (HIV testing[MeSH Terms])) OR (UNAIDS 90:90:90 program[MeSH Terms])) AND (sub Saharan Africa[MeSH Terms])) OR (SSA[MeSH Terms]) OR (Angola)) OR (Benin) OR (Botswana)) OR (Burkina Faso)) OR (Burundi)) OR (Cameroon)) OR (Cape Verde)) OR (Central African Republic)) OR (Chad)) OR (Comoros)) OR (Congo)) OR (Cote d'Ivoire)) OR (Djibouti)) OR (Equatorial Guinea)) OR (Eritrea)) OR (Ethiopia)) OR (Gabon)) OR (The Gambia)) OR (Ghana)) OR (Guinea)) OR (Guinea-Bissau)) OR (Kenya)) OR (Lesotho)) OR (Liberia)) OR (Madagascar)) OR (Malawi)) OR (Mali)) OR (Mauritania)) OR (Mauritius)) OR (Mozambique)) OR (Namibia)) OR (Niger)) OR (Nigeria)) OR (Rwanda)) OR (Sao Tome and Principe)) OR (Senegal)) OR (Seychelles)) OR (Sierra Leone)) OR (Somalia)) OR (South Africa)) OR (Sudan)) OR (Swaziland)) OR (Tanzania)) OR (Togo)) OR (Uganda)) OR (Zambia)) OR (Zimbabwe)	4362

### Study selection and eligibility criteria

#### Study selection

**Fig. 1:**
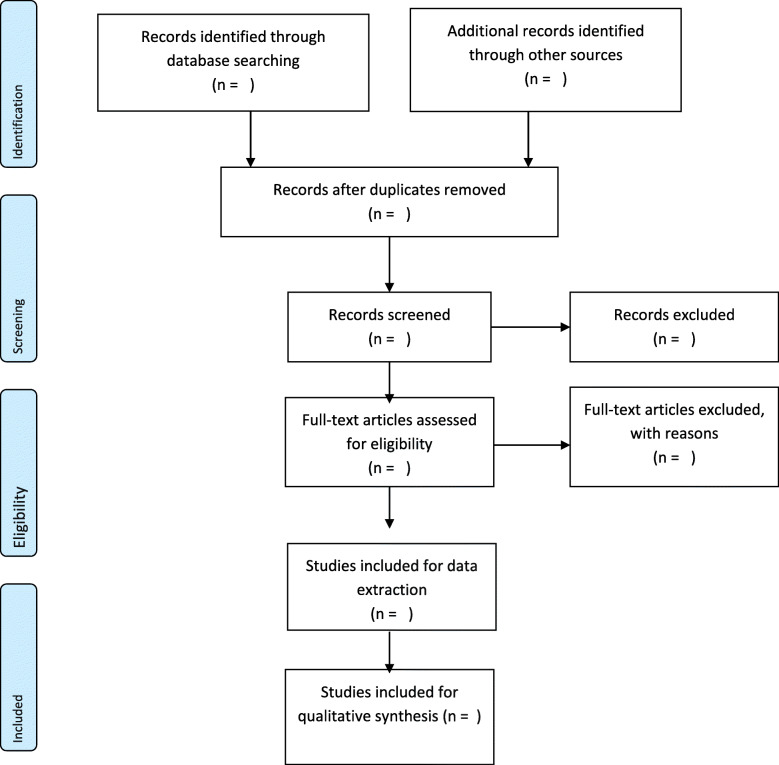
PRISMA flow diagram

Before the abstract and full-text screening phases, the principal investigator (CA) will conduct the keywords search for relevant articles in the electronic databases assisted by PA, BV, and DK. Using the eligibility criteria as a guide, all related titles will be imported onto a Mendeley Desktop library created for this study. At the abstract and full-text screening stages, two reviewers (CA and PA) will use the eligibility criteria to independently sort the studies into two groups—‘include’ and ‘exclude’. Discrepancies between CA and PA responses at the abstract screening stage will be resolved through a discussion by the review team until a consensus is reached. However, discrepancies arising at the full-text screening stage between CA and PA will be resolved by the project supervisor (DK). In the situation where a full-text article cannot be found from the databases, assistance would be sought from the Catholic University’s library or the full text will be requested from the authors via email. Cohen’s kappa coefficient (*k*) statistics between reviewers’ responses will be calculated following full-text screening. Details of the search record (date of search, database, keywords, number of retrievable studies, and number of eligible studies) will be appropriately documented. This study will adopt the PRISMA flow diagram to present the screening results (Fig. 1).

### Eligibility criteria

To ensure the selection of relevant studies for this review, the study selection will be guided by the eligibility criteria as specified under the inclusion/exclusion criteria as specified below.

#### Inclusion criteria

We will include studies that meet the following criteria:

○ Evidence of the study conducted in the SSA

○ Studies that involved HIV key or vulnerable populations

○ Articles that include evidence relating to the first UNAIDS 90 targets

○ Articles that include evidence on HIV status awareness

○ Studies published from 2015 to 2020

○ Studies on knowledge, attitude, and perceptions of the key population on HIV

○ English language publications

○ Primary study designs

#### Exclusion criteria

This study will exclude the following:

○ Studies reporting finding from non-SSA countries

○ Studies conducted before the inception of the UNAIDS 90:90:90 target

○ Publications relating to the second and third UNAIDS 90s targets

○ Publications on the cost-effectiveness of HIV/AIDS interventions

○ Publications in other languages such as French and Arabic

○ Other HIV/AIDS-related review publications

### Charting the data

For this review, the data charting form will be developed for the extraction of all relevant data from the included studies. This form will include the following: author and year of publication, study title, objective/aim of the study, study design, country, study setting, study population, sample size, intervention(s), results, and relevant findings. The data extraction form will be piloted by two independent reviewers (CA and PA) using a random sample of 10% of the included studies to ensure consistency and accuracy. The data extraction form will then be adjusted as required based on feedback from CA and PA. The data extraction will constantly be updated to enable adequate abstraction of all relevant data to answer the review question.

### Collating, summarizing, and reporting of results

We will present information about the included studies that are aligned with this study’s objective. All relevant data extracted from the included studies will be analysed thematically to answer the review question. Thematic content analysis will be conducted to identify relevant themes and sub-themes. Subsequently, these themes and sub-themes will be organized, summarized, and presented as a narrative. Where possible, tables and appropriate figures will also be used to present this study’s findings. This study will mainly focus on outcomes relating to HIV status awareness (for the various included populations). Subsequently, a systematic review and meta-analysis may be conducted using quantitative data if possible.

## Discussion

This systematic scoping review is aimed to map literature and describe the scope of evidence on HIV status awareness, treatment, and viral suppression among HIV key and vulnerable populations as well as among adolescents’ girls and young women since the inception of the UNAIDS 90:90:90 programme in SSA. Although progress has been made towards combating HIV/AIDS globally since the inception of the UNAIDS 90:90:90 programme, the targets are yet to be met even though we are in the last year of its implementation (2020). Hence, evidence is crucial to plan targeted HIV services going forward. This study will be limited to English language publications only due to the lack of expertise for other languages such as French, Arabic, and others which may be a major limitation of this study. This study will also be limited to the first target due to the lack of external funding although the inclusion of the second and third targets would have been great, considering that we are in the last year (2020) of the programme and the targets have not been reached.

Nonetheless, the implications, strengths, and limitations of articles will be adequately reported. The evidence that will be provided by this study may be useful to inform in-country programmes to improve the gains made post-UNAIDS 90:90:90 programme from 2021 onwards. This study also anticipates identifying literature gaps to guide researchers interested in this field of study in the future. The findings of this study will be disseminated through peer review publications, policy briefs, conferences, and media platforms such as local radios.

## Data Availability

All materials used for this review will be duly cited and presented in the form of references.

## Abbreviations

AIDS: Acquired immune deficiency syndrome
ART: Antiretroviral therapy
ARVs: Antiretrovirals
HIV: Human immunodeficiency virus
SSA: Sub-Sahara Africa
UNAIDS: United Nations Programme on HIV/AIDS
WHO: World Health Organization
